# The Significant Effect of Mechanical Treatment on Ceramic Coating for Biomedical Application

**DOI:** 10.3390/ma15196550

**Published:** 2022-09-21

**Authors:** Arman Shah Abdullah, Mohd Nasrun Mohd Nawi, Md Azree Othuman Mydin, Marti Widya Sari, Romisuhani Ahmad, Mohd Mustafa Al Bakri Abdullah

**Affiliations:** 1 Faculty of Technical and Vocational, Universiti Pendidikan Sultan Idris, Tanjong Malim 35900, Perak, Malaysia; 2 Disaster Management of Institute, Universiti Utara Malaysia, Sintok 06010, Kedah, Malaysia; 3 School of Housing, Building and Planning, Universiti Sains Malaysia, Gelugor 11800, Penang, Malaysia; 4 Faculty of Science and Technology, Universitas PGRI Yogyakarta, Yogyakarta 55182, Indonesia; 5 Faculty of Mechanical Engineering Technology, Universiti Malaysia Perlis (UniMAP), Arau 01000, Perlis, Malaysia; 6 Faculty of Chemical Engineering Technology, Universiti Malaysia Perlis (UniMAP), Arau 01000, Perlis, Malaysia

**Keywords:** ceramic coating, physical vapor deposition, wear rate, titanium nitride

## Abstract

Titanium and its alloys are commonly preferred materials used for biomedical implants. However, these alloys have issues related to corrosion resistance as a result of the aggressive attack of human body fluids. Several researchers have attempted to produce a ceramic coating via physical vapour deposition (PVD). A PVD layer consists of pores, pinholes, and columnar growth that attack the substrate as an aggressive medium. The aim of this research is to evaluate the influence of ultrasonic vibration parameters on a TiN-coated biomedical Ti-13Zr-13Nb alloy. This study used TiN to formulate and coat disk-type samples in a fixed condition. Ultrasonic vibration at fixed frequencies was applied to TiN-coated samples for three sets of exposure times. The findings revealed that all TiN-coated samples exposed to ultrasonic vibration had improved corrosion resistance compared to untreated samples. Field emission scanning electron microscopy (FESEM) was employed to analyse sample’s microstructures. The top parameter (16 kHz and 11 min) yielded the most compact coating. Ultrasonic vibration’s hammering effect decreased the size of microchannels in the lining and reduced the rate of corrosion attack. The nanoindentation test showed that coated ultrasonic treated samples had a higher hardness/elasticity (H/E) ratio than untreated samples.

## 1. Introduction

Recently, researchers have become interested in biomaterial due to the high demand for high-performance implants in cardiology, vascular therapy, orthopaedics, trauma, spine, dental, and wound care [1]. Biomaterials are divided into stainless steel, titanium alloys, and CoCrMo. Among these, titanium has characteristics that are suitable for biomedical implants, such as light weight, excellent corrosion resistance, good biocompatibility, and high fatigue strength [2]. However, titanium alloys corrode faster when in contact with body fluid due to the presence of Cr ions. 

Several researchers tried to solve this issue using surface modification methods such as physical vapour deposition [3], chemical vapour deposition [4], thermal oxidation [5], Sol-gel [6], and ion implantation [7]. Previous studies revealed that most researchers used chemical, mechanical, and physical methods to improve corrosion resistance. A few authors used mechanical treatment as an alternative way to improve the performance of mechanical properties or corrosion resistance for coated or untreated samples. For instance, Chenakin et al. applied ultrasonic impact treatment to a biomedical CoCrMo alloy in an artificial saliva solution to enhance corrosion resistance. They reported that surface microhardness could be improved using ultrasonic treatment to increase corrosion resistance compared to as-polished alloys [8]. Tiringer et al. applied grinding and polishing to AA7075-T6, and found that the application improved its corrosion resistance [9]. In addition, Zhang et al. employed wet micro-abrasive blasting as a mechanical treatment to investigate the cutting tool in the machining process. They found that the insert, which was applied via micro-abrasive blasting, had a longer cutting life and a more stable cutting force. This application improved the cutting performance of the insert [10]. Sun et al. applied a mechanical treatment (sandblasting) to aluminium alloy to improve the corrosion behaviour of fibre metal laminates (FMLs). The galvanic corrosion resistance of FMLs was enhanced by sandblasting, micro-arc oxidation, and laser ablation to create a separate layer of aluminium oxide between aluminium and carbon fibre [11]. Recently, Tobola et al. studied the effect of turning slide burnishing on Ti-6Al-4V and found that it caused many defects, such as dislocations and grain boundaries [12]. Mechanical treatments, such as ultrasonic impact treatment, grinding and polishing, wet micro-abrasive blasting, sandblasting, and slide burnishing have been extensively studied. However, ultrasonic vibration, which is a simple method, has not been extensively studied, especially on ceramic coatings, such as TiN. It was expected that the application of ultrasonic vibration to TiN-coated titanium alloy would improve corrosion resistance, and thus, solve the corrosion problem regarding biomaterial in implants. Hence, this study aimed to evaluate the effectiveness of the ultrasonic machining process on ceramic coating (TiN) to improve the corrosion behaviour of Ti-13Zr-13Nb alloy for biomedical implants. This result will produce significant knowledge in terms of explaining how mechanical treatment methods can improve the coated implant materials. 

## 2. Materials and Methods

### 2.1. Material Preparation

Biomedical grade Ti-13Zr-13Nb with a chemical composition (in wt %) of Nb, 14; Zr, 13.5; Fe, 0.05; C, 0.04; N, 0.02; H, 0.002; O, 0.10; and Ti, balanced; was employed. Rods were cut into disks with 10 mm diameters and 2 mm thicknesses using a precision cutter. Before TiN coating, substrates were cleaned ultrasonically for 30 min using acetone, followed by steam cleaning and drying using compressed air. SiC paper was used to mirror polish all substrates. 

### 2.2. TiN Coating via Physical Vapor Deposition

The cathodic arc physical vapour deposition (CAPVD) method was used to coat Ti13Zr-13Nb alloy with TiN. Experiments used 99.99% pure titanium. Before deposition, metal ion etching was applied to the substrates for 5 min at −1000 V bias voltage. 

After that, TiN was used to coat Ti 13Zr-13Nb alloy using the following fixed parameters for the deposition process: cathodic current, 100 A; nitrogen gas flow rate, 300 standard cubic centimetres per minute (SCCM); substrate temperature, 300 °C; substrate bias, −125 V; and 1 h deposition time. 

### 2.3. Mechanical Treatment

The ultrasonic vibration machine introduced a hammering effect on the TiN coating to produce a compact coating. First, the ultrasonic vibration machine was fixed to 16 kHz while exposure time varied from 5 to 11 min. After that, Ti-13Zr-13Nb samples were fixed in a special fixture. The sample was poured with 200 µm steel balls before the ultrasonic treatment was applied. Next, the fixture was mounted on a magnetic table and aligned with the ultrasonic tool. The *z*-axis was secured to avoid excessive load and vibration on the sample. 

### 2.4. Characterization

This study used field emission scanning electron microscopy (FESEM, Supra 35 VP, Carl Zeiss, Jena, Germany) to present surface morphology. A nanoindenter was used to assess the hardness of TiN-coated substrate with and without ultrasonic vibration. A special glue was used to mount the samples to the sample holder, and they were left for one night before indentation testing. To mitigate the effects of gravity, the sample was clamped horizontally to the nanoindentation-testing machine. The penetration load was arranged so that the depth of tip penetration remained 20% lower than the thickness of the coating. During the indentation process, this arrangement prevented the formation of dents on the substrate material. Each sample had five indentations with average values.

Kokubo solution at 37 ± 0.1 °C with a conventional three-electrode cell powered by a potentiostat/galvanostat (Princeton Applied Research Model VersaSTAT 3–300) was used for electrochemical tests. The composition of the Kokubo solution used was Kokubo (c–SBF) Na^+^, 142.0; K^+^, 5.0; Ca^2+^, 2.5; Mg^2+^, 1.5; HCO_3_, 4.2; Cl, 147.8; HPO_4_, 1.0; and SO_4_, 0.5. The counter and reference electrodes were saturated calomel (SCE) and graphite electrodes, respectively. Samples were tested at 0.6667 mV/s. Electrochemical impedance spectroscopy (EIS) was used to measure the corrosion process between the coatings and the substrate. This study used three conventional electrodes, which were similar to the Tafel plot. The Kokubo solution (pH 7.4) was set at 37 °C for all electrochemical procedure tests. A frequency response analyser (FRA) was used to analyse impedance measurements. The frequency range of 10 kHz–1 Hz was used to record the spectrum. The applied alternating potential had a root mean square amplitude of 10 mV on the open circuit potential (OCP).

## 3. Results

**Figure 1:**
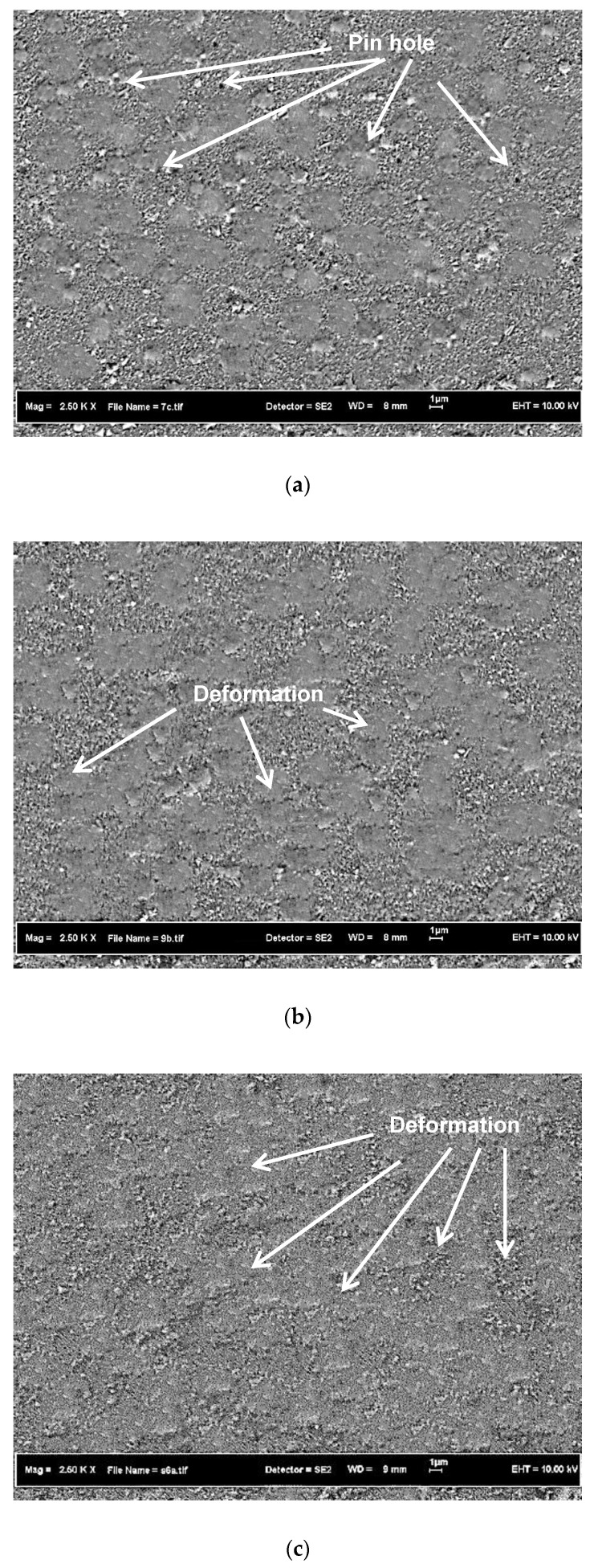
Surface morphology of TiN after ultrasonic treatment at 16 kHz for exposure times of (**a**) 5 min, (**b**) 8 min, and (**c**) 11 min.

Figure 1a–c present SEM micrographs of ultrasonic treatment on TiN-coated Ti13Zr13Nb alloy at several exposure times. Findings reveal the presence of microdroplets on samples without ultrasonic vibration and with ultrasonic vibration for 5 min. However, TiN-coated samples subjected to 8 and 11 min of ultrasonic exposure had no microdroplets. The surface morphology of TiN-coated samples showed significant differences when exposure times increased from 5 to 11 min due to increases in deformation. With longer ultrasonic vibration exposure times, samples’ TiN coatings were penetrated by more steel balls, which increased deformation. Exposure times also had a significant effect on coating thickness, as the coating layer was compressed. In addition, deformation allowed pinholes and channels to fill. Similar compacting behaviour was observed by Bouzakis et al. [13] when sandblasting was used on PVD-coated samples. It is also understood that ultrasonic treatment can remove loose microdroplets that are poorly adhered to a coating’s surface.

**Figure 2:**
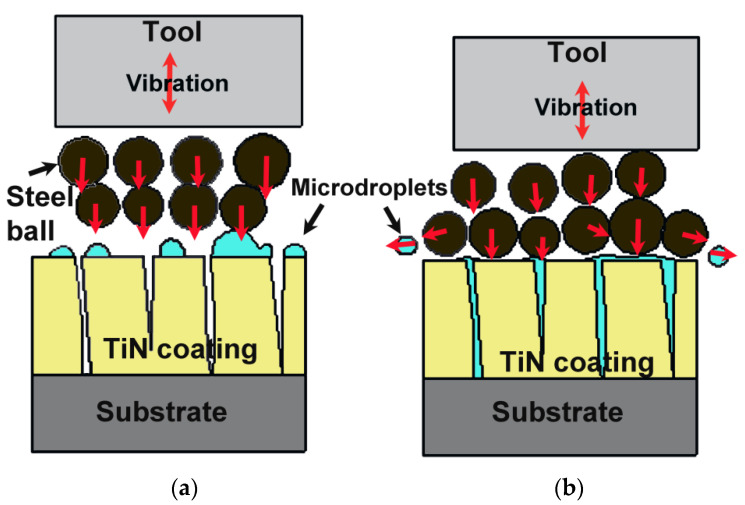
TiN-coated samples (**a**) before and (**b**) after being subjected to ultrasonic vibration.

Figure 2 shows the mechanism of ultrasonic treatments that created a hammering effect using the tool to transfer impact to micro steel balls. When the micro steel balls collided into each other, they vibrated and squeezed the microdroplets and coating surface simultaneously, creating micro deformations. Sticky microdroplets were compressed into microchannels and pinholes, which removed loose microdroplets from the coating’s surface. The coated surface produced more coating deformation when the frequency and exposure time were increased, which led to filling the gaps in pinholes and channels. Bouzakis et al. [13] observed the same compacting behaviour during sandblasting of PVD-coated samples. Similar behaviours were observed when coated samples were treated using the ultrasonic machine. In addition, the ultrasonic machine squeezed some of the microdroplets on coated samples; hence, loose microdroplets were removed using ultrasonic treatment, which reduced the adhesion of microdroplets to the coating’s surface. The coating’s compactness was improved using ultrasonic treatment that led to enhancement of the permeable defects on a PVD-coated sample. This result will continue via a cross-section view to prove the deformation of a coated sample on another site. 

**Figure 3:**
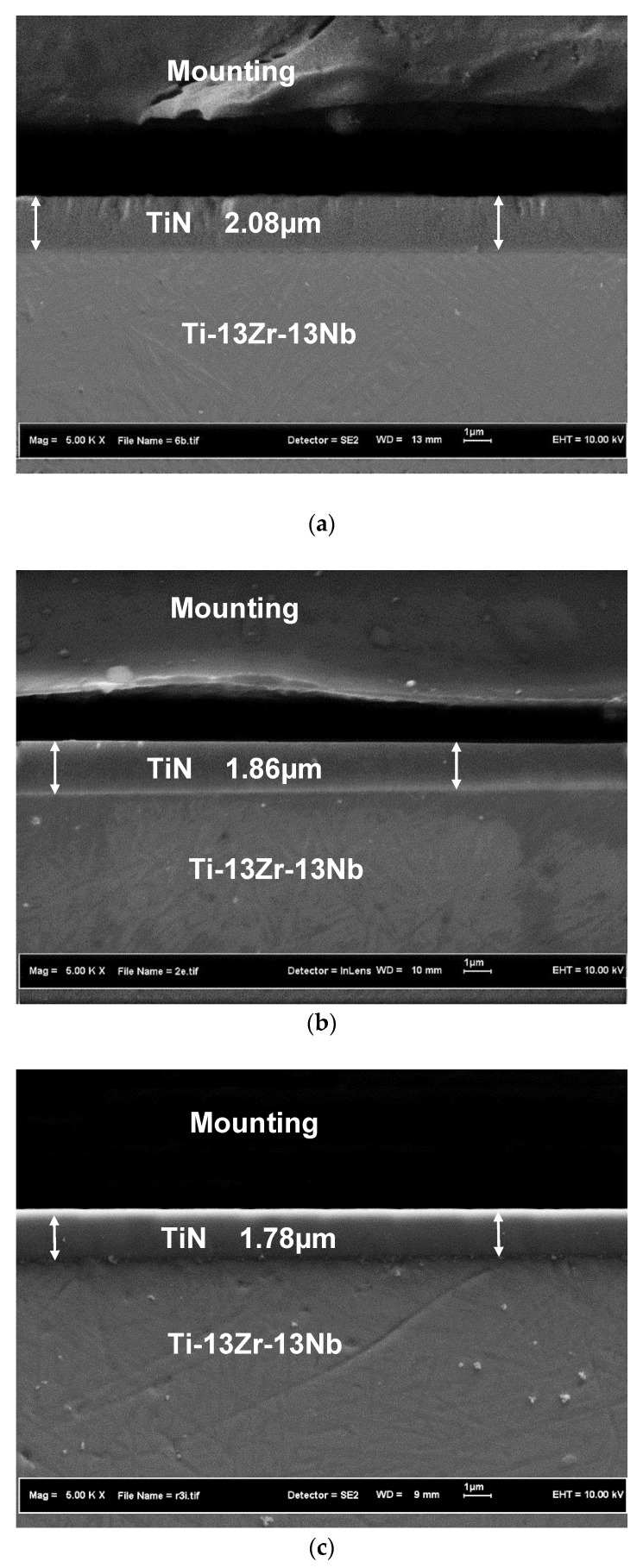
Cross-sectional view of TiN after ultrasonic treatment at 16 kHz for (**a**) 5 min, (**b**) 8 min, and (**c**) 11 min.

**Figure 4:**
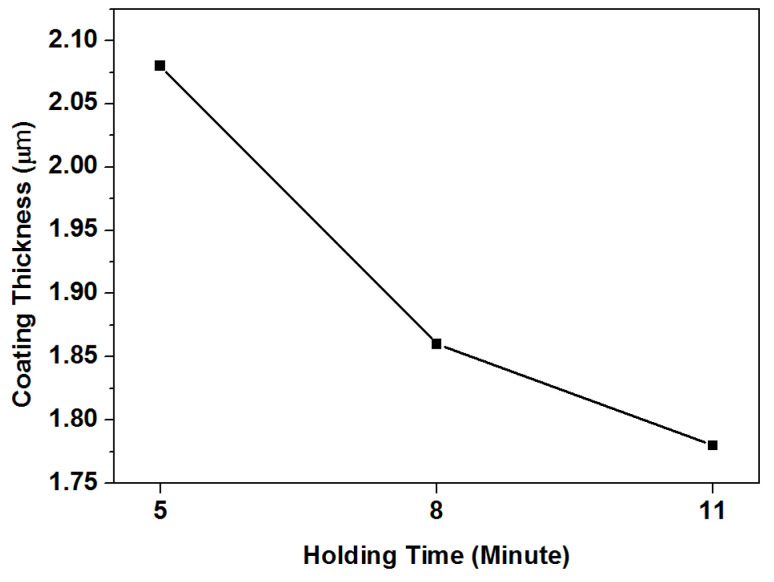
Effect of ultrasonic treatment on TiN coating thickness at 16 kHz for different exposure times.

Figure 3 shows cross-section views of coated samples that underwent ultrasonic treatment at 16 kHz for 5, 8, and 11 min. Coatings had even forms without voids between substrates and coatings, and good adhesion strengths. Figure 4 presents the effect of ultrasonic vibration parameters on coating thickness. Coating thickness decreased approximately 10% from 5 to 8 min, and approximately 4% from 8 to 11 min. It can be concluded that the hammering effect of ultrasonic vibration compacted the TiN coating, which enhanced the impermeable defect of PVD coating. Additionally, the compactness of the coated sample was improved by ultrasonic treatment at extreme low conditions, such as 8 kHz and 5 min. Plastic deformation occurred at the nano level on TiN grains, which transformed into an elongated shape after ultrasonic treatment. Ultrasonic vibration deformed TiN grains and reduced the porosity of the TiN-coated layer. Similar phenomena were reported when coated samples were sandblasted at different blasting pressures and times [13,14,15,16].

**Figure 5:**
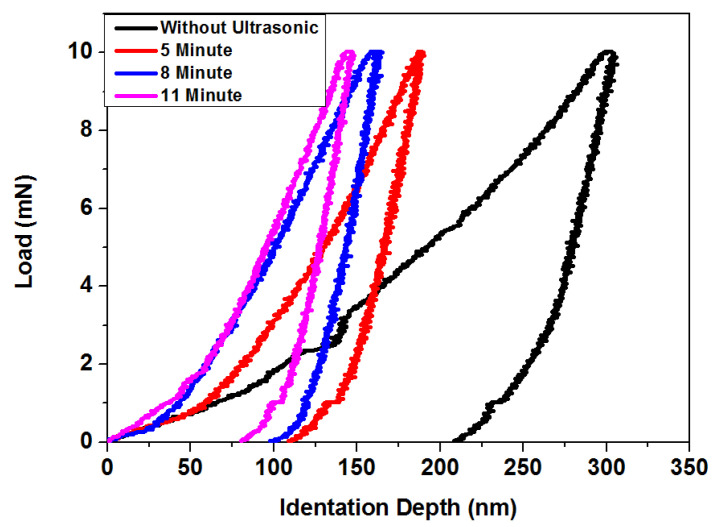
Load vs. displacement curve of TiN-coated samples after ultrasonic treatment at 16 kHz for 5, 8, and 11 min (extreme high condition).

Figure 5 shows the relationship between hardness and exposure times at 16 kHz. Hardness averages were 9.821, 12.12, and 13.10 GPa for 5, 8, and 11 min, respectively. At 11 min, the PVD coated sample’s hardness increased more than three times using ultrasonic treatment. Hardness increased at 5 and 8 min compared to exposure times of 8 and 11 min. Table 1 illustrates the results regarding maximum depth. Exposure times had a strong influence on the hardness and elastic modulus of PVD coating. Table 1 shows the effect of exposure times on H/E. The findings reveal an increase in plastic deformation resistance of coated samples when using ultrasonic vibration. The resistance of the plastic deformation increased from 5 to 8 min and from 8 to 11 min. This confirmed that both ultrasonic frequency and time played active roles in causing deformation of coated layers, which finally increased coating hardness. Longer exposure times and higher ultrasonic frequencies facilitated compression and deformation on the TiN-coated layer [13].

**Figure 6:**
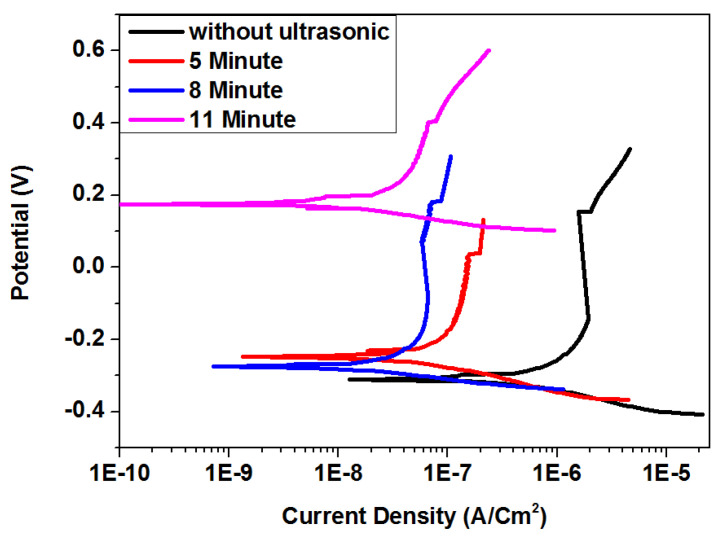
Tafel plots of TiN-coated samples after ultrasonic treatment at 16 kHz for 5, 8, and 11 min (extreme high PVD condition).

Figure 6 shows the anodic and cathodic polarization curves of ultrasonic vibration at various exposure times on TiN coating. The curves for ultrasonic treated TiN-coated samples shifted to the left compared to untreated samples, indicating that treated samples had lower current densities. Similar behaviour was shown for ultrasonic exposure time from 5 to 11 min. These findings reveal that coated samples with longer exposure times had corrosion inhibitors that predominantly controlled anodic reaction. The ultrasonic treatment inhibited the anodic dissolution of titanium alloy and cathodic hydrogen evolution reaction. Table 2 presents output data from Tafel plots, which indicate an approximate 83% decrease in corrosion current density with ultrasonic exposure from 5 to 11 min. This result confirmed the earlier finding for Tafel plots regarding the improvement of corrosion resistance at longer exposure times. Deformation at longer exposure times was more noticeable compared to lower exposure times. SEM micrographs confirm the severity of deformation of coated surfaces at higher exposure times compared to lower exposure times. Generally, current density was inversely proportional to ultrasonic frequency and exposure time. Current density was reduced when ultrasonic frequency and exposure time were increased, despite the extreme conditions of the PVD coating. When current density was decreased, samples and solutions had less current flow. There was a lack of information for the Tafel plot as it only described current flow; it did not explain the mechanism between the solution, the coating, and the substrate. This result was confirmed using EIS data regarding the mechanism after immersing the sample. 

**Figure 7:**
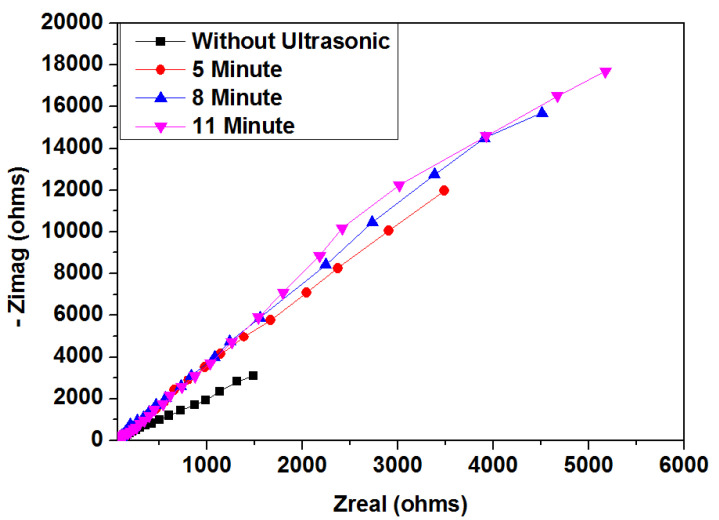
Nyquist plots for TiN-coated samples after ultrasonic treatment at 16 kHz for various exposure times (extreme high PVD condition).

**Figure 8:**
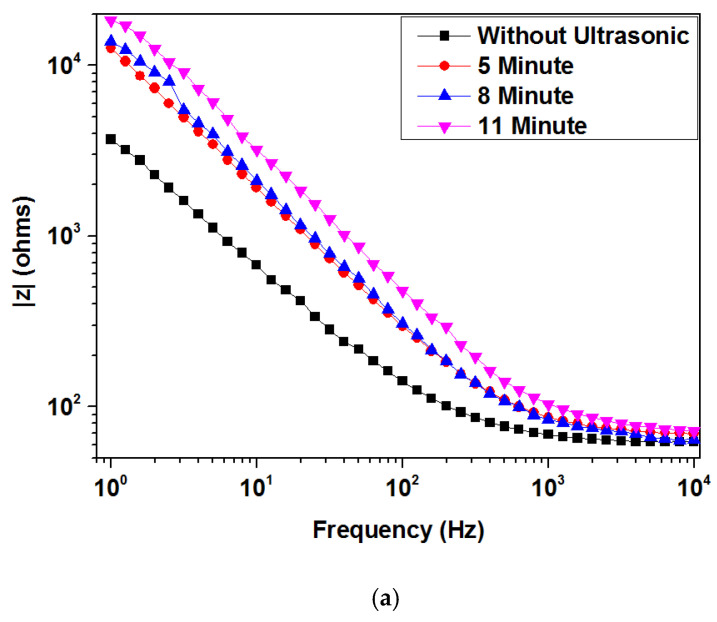

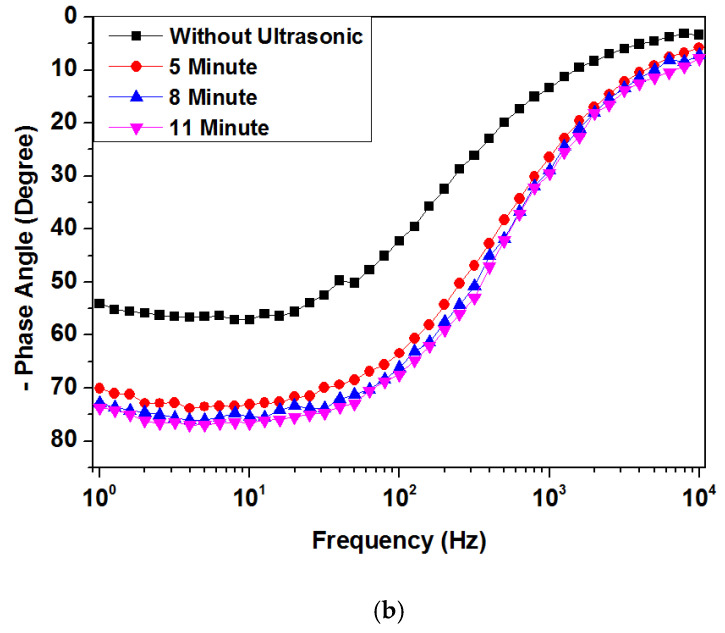
Bode Plots (**a**) log |z| vs. log f and (**b**) phase angle vs. log f for ultrasonic treated TiN coating at 16 kHz for various exposure times.

**Figure 9:**
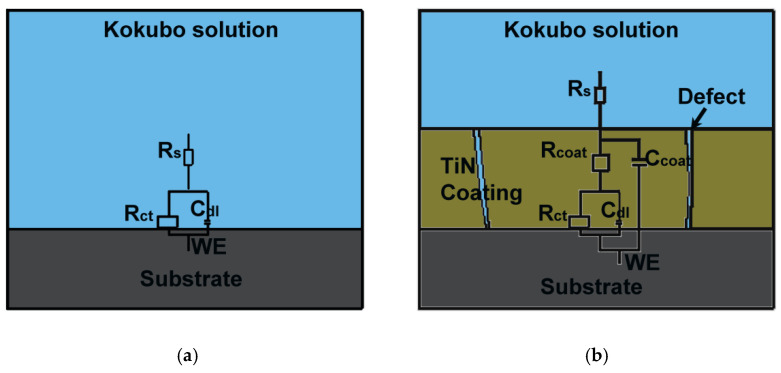
Schematic diagrams representing phenomena that occurred when (**a**) uncoated and (**b**) coated samples were immersed in Kokubo solution, along with their equivalent circuits.

Figure 7 presents a Nyquist plot for TiN coatings after ultrasonic vibration at 16 kHz for various exposure times. Ultrasonic vibration can strongly influence the corrosion resistance of TiN-coated samples at various exposure times. Resistance was higher for coated samples treated with ultrasonic vibration. Additionally, resistance increased for samples treated with ultrasonic vibration when exposure times increased. Longer exposure times caused increases in corrosion resistance. Figure 8a,b show Bode plots (log |z| vs. log f and phase angle vs. log f) for ultrasonic treatment with different exposure times. At 11 min, |z| values were significantly higher than |z| values at 5 and 8 min. Longer exposure times improved corrosion protection. In Figure 8b, the constant appears two times, indicating the interface of the solution/TiN and the TiN/substrate. Table 2 provides EIS output data illustrating that R_ct_ increased approximately 26% from 5 to 11 min. When R_ct_ values and exposure time increased, C_dl_ values decreased, creating a surface film. This phenomenon was discussed in the previous section. Ultrasonic parameters were directly proportional to charge transfer resistance. Increases in ultrasonic vibration and exposure time can increase charge transfer resistance. Increases in ultrasonic frequencies and exposure times enhanced the compactness and defects that prevented the solutions from penetrating the coating and reacting with the substrate, as shown in Figure 9. One time constant can be observed when the uncoated sample was immersed in Kokubo solution. This one time constant was attributed to the resistance of the substrate to the Kokubo solution; the oxide layer on the substrate could not be protected from the aggressive solution attack. The coated sample had two-time constants. Previous studies reported that the equivalent circuit was common for coating samples [17,18,19,20]. The solution and substrate had two interfaces: (1) an interface between the solution and the coated sample, and (2) an interface between the coated sample and the substrate. Coating defects (pore, columnar growth, and porosity structure) permitted the solution to pass through the coating. It attacked the substrate, which lowered the R_ct_ value at lower ultrasonic frequency and exposure times. When ultrasonic parameters were increased, coating samples became more compact, which impeded the solution from passing through the coating and attacking the substrate. 

## 4. Conclusions

This study produced the following conclusions:

I. Ultrasonic frequency and exposure time directly affected the hardness of TiN-coated substrate. Increases in the TiN coating’s hardness were almost linear when both ultrasonic frequency and exposure time were increased. In contrast, the effect on the TiN coating’s thickness was enhanced by two ultrasonic parameters. Increased ultrasonic frequency and exposure time reduced the two responses; they compacted the coating layer from micro to nanoscale levels. The evidence shows a high elastic recovery in load vs. displacement curves.
II. Current density has an inverse effect on vibration frequencies and exposure time. Increased vibration frequency and exposure time reduced current densities of ultrasonically treated TiN-coated substrates. Pores and voids in TiN coatings were reduced when the vibration frequency and exposure time were increased, which led to an increased resistance to charge transfer Rct. Compared with the untreated sample, reducing Icorr values in ultrasonically treated TiN-coated substrates improved corrosion resistance.

## Figures and Tables

**Table 1:** Summary of output data from nanoindentation tests for ultrasonic treated samples at 16 kHz for different exposure times (extreme high PVD condition).

Sample Conditions	Maximum Depth (nm)	Hardness (GPa)	Elastic Modulus E (GPa)	H^3^/E^2^ Ratio
**Without Ultrasonic**	306.315	3.871	121.316	0.0039
**5 min,** **16 kHz**	190.235	9.821	242.640	0.0161
**8 min,** **16 kHz**	165.213	12.122	256.998	0.0270
**11 min,** **16 kHz**	150.012	13.101	263.221	0.0325

**Table 2:** Corrosion parameters calculated from Tafel and EIS results for ultrasonic treatment of TiN coatings at 16 kHz for different exposure times.

Sample Conditions	Ecorr (mv)	Icorr (µA/cm^2^)	Corrosion Rate (mm/Year)	Rct	Cdl
**Without Ultrasonic**	−311.92	0.421	8.8354 × 10^−3^	2536	1.685 × 10^−5^
**5 min, 16 kHz**	−282.17	0.065	1.3152 × 10^−3^	7505	1.952 × 10^−6^
**8 min, 16 kHz**	−122.07	0.0363	0.726 × 10^−3^	8950	0.3448 × 10^−6^
**11 min, 16 kHz**	174.21	0.0100	0.2250 × 10^−3^	9500	3.248 × 10^−7^

## Data Availability

Data presented in this study are available on request from the corresponding author.
